# Regeneration Abilities among Extant Animals Depend on Their Evolutionary History and Life Cycles

**DOI:** 10.3390/jdb12010008

**Published:** 2024-02-09

**Authors:** Lorenzo Alibardi

**Affiliations:** 1 Comparative Histolab Padova, 35100 Padova, Italy; lorenzo.alibardi@unibo.it; 2 Department of Biology, University of Bologna, Via Selmi 3, 40126 Bologna, Italy

**Keywords:** metazoans, healing, regeneration, scarring, evolution, genes

## Abstract

The present brief manuscript summarizes the main points supporting recently proposed hypotheses explaining the different distributions of regenerative capacity among invertebrates and vertebrates. The new hypotheses are based on the evolution of regeneration from marine animals to the terrestrial animals derived from them. These speculations suggest that animals that were initially capable of broad regeneration in the sea underwent epigenetic modifications during terrestrial adaptation that determined the loss of their regenerative abilities in sub-aerial conditions. These changes derived from the requirements of life on land that include variable dry and UV-exposed conditions. Terrestrial conditions do not allow for organ regeneration, especially in arthropods and amniotes. Nematodes, the other main metazoan group unable of regeneration, instead evolved eutely (a fixed number of body cells), a process which is incompatible with regeneration. All these changes involved gene loss, modification and new gene interactions within the genomes of terrestrial adapting animals that gave rise to sophisticated invertebrates and vertebrates adapted to living on land but with low cellular plasticity.

## 1. Introduction

The ability to recover after injury in adult animals is very variable; from scarring to an extended healing process, to large organ-appendage regeneration, to whole body regeneration, with the latter only being present in invertebrates living in water (marine and some freshwater species). The present brief review synthesizes new hypotheses on animal regeneration and speculations based on evolutionary considerations. The latter have been previously broadly discussed in regard to the distribution of regeneration among invertebrates and vertebrates [1,2,3,4,5,6,7,8]. In these papers, the reader can find numerous references on animal regeneration, often mainly based on cellular and molecular studies. In contrast, the present brief summary refers to speculations about evolution that were recently proposed by the author. These hypotheses follow the points indicated here: (1) life initiates in the sea and regeneration was common in primitive forms; (2) asexual reproduction, broad metamorphosis and regeneration only occur in saltwater and sometimes also in freshwater animals; (3) regeneration from an adult body variably re-utilizes developmental genes, especially in simpler rather than in complex animals; (4) the evolution of eutely in aschelminthes erased regeneration; (5) adaptation to the land determined epigenetic changes that eliminated larval forms, broad metamorphosis and, consequently, also regeneration; (6) land-adapted larvae and their metamorphoses were simplified (molting), a process that abolished regeneration; and (7) recovery after injury in terrestrial animals varies from a generalized scarring to occasional broad heteromorphic healing and/or *regengrow*.

## 2. Point 1

**Figure 1:**
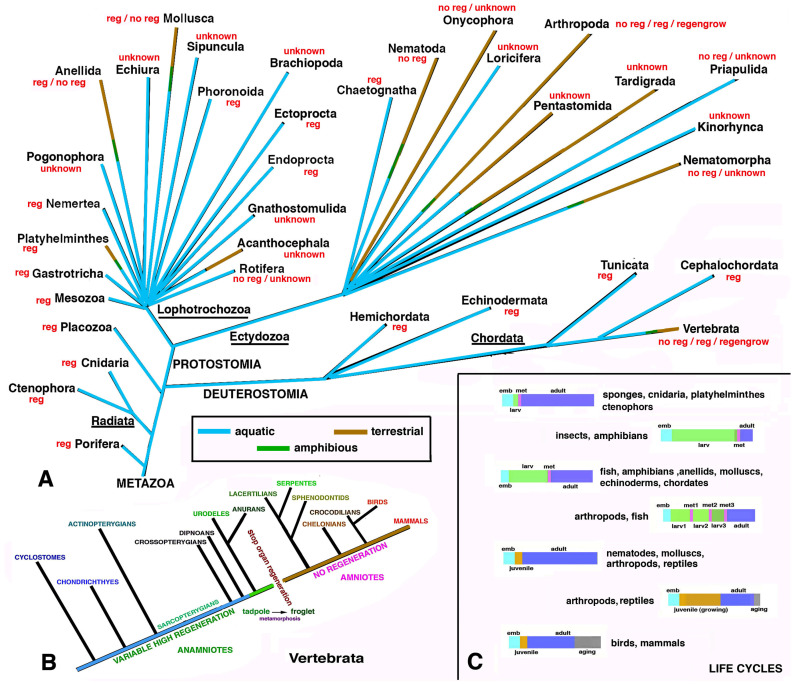
Schematic drawing summarizing some of the hypotheses presented here. (**A**) Animal phyla with presence of most regenerative (reg), non-regenerative (no reg) or species with unknown regeneration. Most aquatic species (light blue lines) can regenerate vast body parts, from morphallaxis to epimorphosis. Terrestrial species (brown lines) only repair their tissue by non-functional scarring, partial and heteromorphic healing (“regeneration”) or by *regenegrow*. (**B**) Detail on vertebrate groups showing that only anamniotes living in water or amphibious (different fish and numerous amphibians) present broad regenerative ability. In contrast, terrestrial vertebrates (amniotes) have limited or absent regenerative ability. (**C**) Colored bars representing different stages of the life cycles in different animals, from the embryo to the adult phase. Animals with one or more metamorphic transitions (pink area, indicated with met) can regenerate large or small organs after their loss. While arthropods have land-adapted larvae or juveniles and a limited metamorphosis, amniotes do not possess a larval phase, which is replaced with a variable long juvenile phase with no metamorphosis (see text and references for more details).

Numerous unicellular animals, the initial life forms that originated over 1.5 billion years ago in the sea, can regenerate their damaged cell. Also, the lower forms of invertebrates that appeared in the Precambrian period (sponges, cnidarians ctenophorans and platyhelminthes) can broadly regenerate. This process occurs through two main modalities: (1) through whole-body regeneration (morphallaxis) where cells from small body fragments re-associate and re-pattern into a new, initially smaller, animal, or (2) by a polarized regeneration with the formation of an outgrowth that progressively grows, reforming the lost organ/part of the body (epimorphosis). As a basic life characteristic, regeneration ability was/is a general property of animal tissues. Ancient, but more recent, Cambrian–Ordovician evolved phyla (aschelminthes, anellids, molluscs, echinoderms, lower chordates and some other minor phyla) can variably regenerate after injury or organ loss. In these animals, regeneration is also relatively frequent, from a broad body or organ regeneration to a variable degree of tissue healing. Only terrestrial-adapted species (some nematodes-nematomorpha, most arthropods and amniotes) do not regenerate or heal through limited wound healing. The term “regeneration”, when used for fully terrestrial animals (e.g., digit or skin regeneration in some mammals, organ regeneration during embryogenesis or fetal life, leg regeneration in arthropods, tail regeneration in some reptiles, etc.), is actually a variable process of wound healing. It does not completely restitute the original form or function, and cannot re-pattern the lost structure, and it is mainly tissues that are regenerated. This process has previously been termed *regengrow*, and indicates that *regen*eration takes place together with somatic *grow*th and/or during molting (particularly in arthropods). The extensive healing associated with growth often occurs over a long period and cannot be repeated after full somatic growth or the last molt. Embryonic and fetal “regeneration” also occurs at the same time as the growth of embryos or fetuses, and this contemporaneous “regeneration and growth” represents *regengrow*. However, terrestrial animals generally heal their wounds or organ loss through scarring, a fast cell process that impedes extensive life-threatening dehydration and microbial invasion. During the specific environmental adaptations in different animals, especially terrestrial, the initial regeneration present in aquatic species was lost or became limited. The distribution of regeneration versus scarring or a limited healing between aquatic (marine and freshwater) and terrestrial animals is summarized in Figure 1A,B.

## 3. Point 2

Broad regeneration only occurs in species that demonstrate asexual reproduction and often large, metamorphic transformations from a larval form to the adult form during their life cycles (anellids, some molluscs, echinoderms, tunicates, fish, amphibians, etc., Figure 1C). These are exclusively aquatic animals (mainly marine but also some freshwater or amphibious) with highly hydrated bodies and an aquatic dispersion of embryos and larvae. As such, the dispersion of soft-hydrated larvae in a terrestrial environment is not possible (see below). Therefore, during evolution towards terrestrial conditions, the, originally marine, animals modified their life cycle, erasing the larval stage and indirect development in favor of direct development, to be better adapted to the land. This developmental adaptation, however, eliminated their ability to regenerate (see below). Since numerous freshwater-adapted animals were derived from previous terrestrial species with no regeneration ability, their regeneration remained limited.

## 4. Point 3

Genomes of animals (aquatic) that include genes which operate during asexual reproduction or metamorphic transitions can variably re-utilize these for regeneration in adults. However, developmental genes activated during embryogenesis cannot be utilized in the same sequence for regeneration in most animals. In fact, while embryogenesis initiates from a single fertilized cell, regeneration begins within an adult body composed of numerous differentiated cells that influence the new cells formed for regeneration. It is, therefore, impossible that, even in the lower whole-body regenerative competent animals, regeneration activated the same sequence of developmental genes as used during embryogenesis. As such, genomes of species that do not include larval forms and broad metamorphic transformations (nematodes, many arthropods and amniotes) have lost or deactivated the genes involved in regeneration. In fact, the formation of a hydrated blastema, destined to rapidly dry out on land, is incompatible with terrestrial life. Although some terrestrial-adapted larvae, such as those of holometabolous insects, experience an apparent broad metamorphosis from the pupa to the adult, this process derives from limited regions indicated as imaginal disks. These (stem) cells replace or assimilate with those of the intermediate pupa stage, and appendage regeneration during molting re-uses these cells.

## 5. Point 4

The evolution of eutely in aschelminthes (nematodes, nematomorphs, rotifers, gastrotrichs, achantocephalans, kynorhynchs) eliminates their ability to regenerate tissues or organs after injury. Eutely is a phenomenon present in animals composed of a specific and invariant number of cells. In particular, in the best-known nematodes, a stereotypic form of embryonic development gives rise to animals formed by a fixed number of cells. Numerous species, blastomeres (embryonic dividing cells) undergo a large DNA-elimination processes (with the exception of cells in the germinal line). This DNA loss and extremely stereotypical differentiation determines the lack of regenerative capacity in these small animals, particularly in terrestrial-adapted species.

## 6. Point 5

The importance of sensing the surrounding environment with derived epigenetic changes in the genome of animals (DNA and histone methylation, ncRNAs and transposone changes) is increasingly being recognized as driving broad evolutionary adaptations. In particular, epigenetic effects might have influenced genes involved in development and regeneration that were expressed in a marine environment. These genes were of no use in a dry, UV-exposed and microbial-load terrestrial condition, primarily because no highly hydrated regenerating organ can resist desiccation. Therefore, some genes involved in regeneration underwent a negative selection, erasing the ability for regeneration in land-adapting animals. This process mainly occurred during the evolution of terrestrial arthropods (insects, myriapods, arachnids, etc.) and amniotes (reptiles, birds and mammals). The identification of specific developmental genes that were epigenetically altered during the terrestrial evolution of animals may represent a fertile field for future investigations. The loss of regenerative plasticity was countered by the evolution of the adaptive immune system, large brains and complex cognitive abilities and behaviors that help these animals to avoid injuries.

## 7. Point 6

While marine dispersion of larvae is common in a liquid medium, this is not possible on solid terrain. Therefore, larvae and intense metamorphic transformations (destruction of larval organs that were reformed de novo as adult organs) were erased from the life cycle of terrestrial invertebrates and vertebrates. New forms of reproduction and development were acquired in invertebrates and vertebrates that became terrestrial, and the loss of larvae–metamorphosis determined their incapability to regenerate organs. Also, in holometabolous insects with broad metamorphic changes, the terrestrial environment did/does not favor extensive regeneration. Insect metamorphosis is the replacement of organs from imaginal disks and does not require extensive organ destruction and regeneration into adult organs, except in appendages (that can regenerate during molting). Eggs adapted for direct development, in terrestrial invertebrates or the amniotic egg and later placentation in amniotes, avoid exposing the embryo to a dry environment that would stop survival on land. During the water-to-land transition in invertebrates and vertebrates, at least some developmental genes for the formation of larvae and for metamorphosis were altered, lost or inserted in different gene networks. This loss erased the ability to regenerate in favor of scarring. Few terrestrial species recover from large injuries; they sometimes broadly heal and variably “regenerate”, but often they actually *regengrow* heteromorphic structures (limbs, tails, liver, ear damages, spinal cord, etc.). During scarring, extensive wound healing, heteromorphic regeneration or *regengrow*, only a few developmental genes can be re-utilized.

## 8. Point 7

As indicated above, true regeneration on the land is very difficult, and most fully terrestrial animals, invertebrates and vertebrates cannot regenerate their organs but instead undergo limited tissue healing which mainly results in scarring. The term regeneration is customarily utilized for a different process of healing in animals, including terrestrial species, without considering the period of the life cycle in each different species (Figure 1C). True regeneration versus wound healing associated with somatic growth (*regengrow*) represents different processes. True regeneration restitutes a large part of or complete organs and their function and occurs in a short time frame in comparison to the life cycle (2–4% of the total lifetime span). Regeneration progresses independently from somatic growth or molting and can be repeated at almost any age. Regeneration evokes a patterning process and not only disorganized and volumetric tissue formation. The so-called regeneration of embryonic or fetus organs in juveniles still in their growth course is, therefore, not really regeneration but instead is *regengrow*. This means that one cannot distinguish, at the end of the process, what is derived from the process of growth and what instead derives from the healing (“regeneration”) after injury or amputation. *Regengrow* cannot occur after the end of somatic growth in amniotes or after the last molt in arthropods where scarring is the only output.

In conclusion, true regeneration only occurs in aquatic invertebrates, especially marine animals, that are able to restructure their entire body (morphallaxis or whole-body regeneration) or various organs by epimorphosis (polarized regeneration). Among vertebrates, anamniotes including numerous fish and amphibian species possess broad, although more limited, regeneration abilities in comparison to invertebrates. This is also due to their higher biological complexity since gene activation in their injured adult bodies cannot recapitulate the gene activation present during their development. Terrestrial species of invertebrates and vertebrates can only variably heal their wounded tissues, organs or lost appendages through extensive healing or by *regengrow*, but mainly by non-functional scarring. As a counterpart, terrestrial species with refined behaviors are more efficiently capable of avoiding large injuries.

## Data Availability

Not applicable.
